# Inherited Real Risk of Type 2 Diabetes Mellitus: bedside diagnosis, pathophysiology and primary prevention

**DOI:** 10.3389/fendo.2013.00017

**Published:** 2013-02-26

**Authors:** Sergio Stagnaro, Simone Caramel

**Affiliations:** Department of Advanced Research, International Society of Quantum Biophysical SemeioticsLancenigo, Treviso, Italy

## Introduction

Insulin resistance and insulin-secretion derangement are correlated in a stable manner and both play a pivotal role in the onset of Type 2 Diabetes Mellitus (T2DM). In fact, e.g., insulin-secretion is physiologically ruled by insulinemia, by means of a feed-back mechanism, through insulin-receptors localized on the membrane of insular β-cell: the two factors are strictly related each other at large number of different levels. Due to these reasons the clinical on-set of T2DM appears later, because insulin-resistance in liver (Zenda et al., 2003), adipose cells of the abdomen wall and skeletal muscles during the initial stage, i.e., for years or decades, may be balanced by increasing of the insulin secretion. It is generally admitted that the T2DM may occur at a flurry number of years before the clinical diagnosis is made. Importantly, during the time that diabetes is undiagnosed and untreated, “complications,” that could be avoided, are developing. With the aim of an early pre-metabolic diagnosis we introduce a clinical method to assess the Inherited Real Risk (IRR) of T2DM by means of Quantum Biophysical Semeiotics (QBS), which is an extension of medical semeiotics (Stagnaro-Neri and Stagnaro, 2004), interpreting an awful number of QBS signs for diagnostic purposes. The key of this new discipline is the awareness that human bodies are a continuum of biological systems whose dynamics follow the laws of deterministic chaos, which can be measured by means of nonlinear statistical invariants.

## QBS method: IRR of T2DM diagnosis

QBS method allows the clinical and pre-clinical diagnosis of the most severe diseases, e.g., solid and liquid forms of cancer and coronary heart diseases, as well as the IRR of T2DM; i.e., through the auscultatory percussion of the stomach (Stagnaro and Stagnaro-Neri, 1986). Doctors can now evaluate, with a stethoscope and the auscultation of any viscera (i.e., stomach, ureter), mitochondria functions as well as the behavior of any biological system.

As regards the technical procedure, doctors can recognize QBS “Diabetic Constitution” in the easiest way by an “intense” skin pinch at the level of the VI thoracic dermatome. In a healthy patient, “simultaneously” the gastric aspecific reflex is absent, appearing after 24 s sharp (Stagnaro and Stagnaro-Neri, 2004).

On the contrary, in patients predisposed to diabetes, the reflex appears “simultaneously” (positive Siniscalchi's sign), showing an intensity inferior to 1 cm, while in the diabetic patient it is 1 cm or more, in relation to the seriousness of underlying disorder.

Among the different, consistent each others, QBS signs useful for the diagnosis of “Diabetic Constitution” and IRR of T2DM such as, i.e., the pancreatic gastric aspecific reflex (Stagnaro and Caramel, 2013), we illustrate briefly the Osteocalcin test. This original manoeuvre, based on a singular activity of osteocalcin, is reliable in bedside detecting diabetes in 1 min, with the aid of a stethoscope (Stagnaro and Stagnaro-Neri, 1986). In fact, osteocalcin, a product of osteoblasts, among other action mechanisms, stimulates both insulin secretion and insulin receptor sensitivity.

As a consequence, osteocalcin, secreted by the bone cells during mean-intense lasting digital pressure—for instance—applied upon lumbar vertebrae, brings about increasing pancreatic diameters, i.e., technically speaking, type I, associated, Langherans islet microcirculatory activation. By this way, doctors assess pancreas size augmentation, which in health, lasts 10 s exactly. After that, pancreas diameters return to its basal value for 3 s. The second pancreas size increasing lasts 20 s, and finally the third one shows the highest value: 30 s. On the contrary, in case of diabetic constitution the first pancreas increasing persists normally (10 s), but both the second and the third dilations are less than the physiological one (i.e., less than 20 s and respectively 30 s). In presence of intense IRR of T2DM, such as impairment is greater. Finally, in case of T2DM the alteration is present already in the first evaluation, wherein duration appears less than 10 s, inversely related with disorder seriousness.

As a matter of fact, pre-hypertension during young adulthood may be involved by coronary calcium later in life exclusively in presence of IRR of CAD, typical for individuals with lithiasic constitution, present in about 50% of all cases of pre-metabolic and metabolic syndrome (Caramel and Stagnaro, 2012). Considering the frequent association between hypertension and diabetes, it is important, to recognize, bed-side, the diabetic predisposition, now-a-days possible from birth, utilizing a lot of clinical methods, some of them above mentioned.

## Pathophysiology of T2DM: the first five stages

According to clinical and experimental evidences provided by QBS signs and clinical diagnosis (Stagnaro and Stagnaro-Neri, 1986, 2004; Stagnaro-Neri and Stagnaro, 2004; Caramel and Stagnaro, 2012; Stagnaro and Caramel, 2013) the pathophysiology of T2DM can be resumed in five temporal stages, which play a key role in early diagnosis to avoid complications and glucose metabolism impairment:

1. diabetic and dyslipidemic constitution, IRR of T2DM — overt, latent or potential (individual's birth);
2. abnormal synthesis of perivascular GAGs by fibroblasts, pericytes, mioblasts, megacariocytes, a.s.o.; Amiline in the Interstitial Fundamental Substance, glycocalix malfunction in both beta-cells and peripheral target-organ cells. Location: Capillaries, Small Arteries, Arterioles, AVA type II, group B, cutaneous, Endoarteriolar Blocking Devices (EBDs)., a.s.o. (under 10 years old);
3. IRR of T2DM, Microalbuminurie, Initial ATS Plaques, a.s.o. (second decade of life);
4. pre-diabetes, overt microvascular complications — OGTT, Iper-Insulinemic-Normo-Glicemic Clamping, Insulinemia (about third decade of life);
5. Overt T2DM.

Other authors (Weir and Bonner-Weir, 2004) introduced the idea of five stages in the pathophysiology of T2DM. Their approach is more about glucose metabolism and the interplay between beta cell function and insulin resistance, while the one (Stagnaro, 2011) here mentioned is focused on complications.

Since their birth (first stage) all diabetic individuals show QBS signs typical of dyslipidemic “and” diabetic constitutions, and all the related IRR, based on a mitocondrial functional cytopathy termed Congenital Acidosis Enzimo-Metabolic Histangiopathy (CAEMH) (Stagnaro-Neri and Stagnaro, 2004; Caramel and Stagnaro, 2012; Stagnaro and Caramel, 2013), subsequently evolved first into pre-metabolic syndrome (Stagnaro and Singh, 2009) and later on into metabolic one, under the negative influence of well-known environmental factors. In fact, not “all” the individuals, even though obese and/or hypertensive, are at risk of diabetes mellitus, with the same probabilities. On the contrary, the individuals with diabetic IRR are all those who are positive to dyslipidemic “and” diabetic QBS constitutions, inherited mostly from the mother, and associated to the diabetic IRR, *conditio sine qua non* of T2DM.

The second stage of evolution to overt T2DM is characterized by the enlargement of interstitial space, for instance in Langherans's islets, caused by abnormal synthesis of local GAGs, when the production of jaluronic acid appears particularly compromised, showing an altered affinity for water. So that the relation *free water/bound water* appears abnormally high. ATS plaques appear from the second decade of life (third stage). The last two stages, pre-diabetes and overt T2DM, all well clinically known.

The objective QBS examination allows physicians to bedside recognize and quantify, in a few minutes, the presence of the IRR of diabetes or overt T2DM, even initial, through the evaluation of several semeiotics signs, i.e., assessing vasomotility and vasomotion impairments, and the typical pathological EBDs (Ditzel, 1968; Stagnaro and Stagnaro-Neri, 1986, 2004; Stagnaro-Neri and Stagnaro, 2004; Caramel and Stagnaro, 2012; Stagnaro and Caramel, 2013). The IRR of T2DM is characterized by a microcirculatory remodeling due to the functional and structural abnormalities just mentioned.

For instance, in the normal Langheran's islets microcirculatory bed, there are exclusively “normal” type II (present in arterioles, according to Hammersen), but not type I (present in small arterioles) EBDs, i.e., first and second class EBDs (Curri, 1968; Ditzel, 1968). In health, i.e., not involved by Diabetic Constitution, we do not observe type I, subtype b) aspecific, newborn- pathological EBDs in the above-mentioned biological system.

On the contrary, in individuals involved by diabetic constitution as well as diabetic IRR and overt T2DM, in its diverse stages, we observe also newborn-pathological, type I, subtype b) aspecific, EBDs, facilitating the diagnosis and consequently diabetes primary prevention. In addition, the evaluation of Insulin Secretion Acute Pick (ISAP) Renal Test (Stagnaro-Neri and Stagnaro, 1997) is significantly impaired, corroborating the clinical diagnosis.

## T2DM primary prevention

The QBS method was used to monitor tissue acidosis before and during preventive therapies (Stagnaro, 2002) such as “Modified Mediterranean Diet” (a greater amount of fish, i.e., protein and Omega-3 fatty acids, than the normal Mediterranean Diet), CoQ10, melatonin conjugated with adenosine and carnitine (Stagnaro-Neri and Stagnaro, 1996) in T2DM primary prevention and therapy. The QBS therapeutic monitoring corroborates the efficiency of the combination of these treatments for preventive purposes as stated by the reduced level of tissue acidosis, the improvement of tissue oxygenation (Ditzel and Standl, 1975) and mitochondrial activity. Recent clinical experiments done by means of a quantum therapy able to capture and re-transmit customized Extremely High Frequencies (EHF) millimeter waves from pancreatic trigger points evidence an improvement of mitochondrial and endothelial function, showing genetic feedbacks able to heal the “Diabetic Constitution.”

## Current and future developments

Clinical and experimental data collected by QBS diagnosis evidence a patho-physiology of T2DM resumed with first five stages. The presence of the IRR of T2DM, evolution of a congenital “Diabetic Constitution,” is clinically diagnosed from birth, so that an intelligent prevention strategy can be implemented only on those at real risk, especially under a very early diagnosis, done on a large scale in the first decade of life.

## References

[B1] Caramel S. Stagnaro S. (2012). Vascular calcification and Inherited Real Risk of lithiasis. Front. Encocrin. 3:119 10.3389/fendo.2012.00119 23060859 PMC3464397

[B2] Curri S. B. (1968). Le Microangiopatie. Milano: Inverni della Beffa

[B3] Ditzel J. (1968). Functional microangiopathy in diabetes mellitus. Diabetes 17, 388–397 4870944 10.2337/diab.17.6.388

[B4] Ditzel J. Standl E. (1975). The problem of tissue oxygenation in diabetes mellitus. I. Its relation to the early functional changes in the microcirculation of diabetic subjects. Acta Med. Scand. Suppl. 578, 49–58 239527

[B7] Stagnaro S. (2002). Diet and risk of type 2 diabetes. N. Engl. J. Med. 346, 297–298 11807161 10.1056/NEJM200201243460418

[B5] Stagnaro S. (2011). A Clinical Way in Fighting the Five Stages of Type 2 Diabetes Mellitus. Available online at: http://www.biomedcentral.com/1741-7015/9/76/comments

[B6] Stagnaro S. Caramel S. (2013). The role of modified mediterranean diet and quantum therapy in type 2 diabetes mellitus primary prevention. JPNAS 3, 59–70 10.1007/s12603-013-0435-7 PMC12878279 24402398

[B8] Stagnaro S. Singh R. B. (2009). Influence of nutrition on pre-metabolic syndrome and vascular variability syndrome. Open Nutr. J. 2

[B9] Stagnaro S. Stagnaro-Neri M. (1986). Valutazione percusso-ascoltatoria del Diabete Mellito. Aspetti teorici e pratici. Epat. 32, 131

[B10] Stagnaro S. Stagnaro-Neri M. (2004). Le Costituzioni Semeiotico- Biofisiche. Strumento clinico fondamentale per la prevenzione primaria e la definizione della Single Patient Based Medicine. Roma: Travel Factory

[B11] Stagnaro-Neri M. Stagnaro S. (1996). Sindrome di Reaven, classica e variante, in evoluzione diabetica. Il ruolo della Carnitina nella prevenzione del diabete mellito. Il Cuore 6, 617

[B12] Stagnaro-Neri M. Stagnaro S. (1997). Semeiotica Biofisica: valutazione clinica del picco precoce della secrezione insulinica di base e dopo stimolazione tiroidea, surrenalica, con glucagone endogeno e dopo attivazione del sistema renina-angiotesina circolante e tessutale. Acta Med. Medit. 13, 99

[B13] Stagnaro-Neri M. Stagnaro S. (2004). Introduzione alla Semeiotica Biofisica. Il Terreno Oncologico. Travel Factory: Roma Availabe online at: http://www.travelfactory.it/semeiotica_biofisica.htm

[B14] Weir G. C. Bonner-Weir S. (2004). Five stages of evolving beta-cell dysfunction during progression to diabetes. Diabetes 53 Suppl. 3, S16–S21 15561905 10.2337/diabetes.53.suppl_3.s16

[B15] Zenda T. Murase Y. Yoshida I. Muramoto H. Okada T. Yagi K. (2003). Does the use of insulin in a patient with liver dysfunction increase water retention in the body, i.e. cause insulin oedema? Eur. J. Gastroenterol. Hepatol. 15, 545–549 10.1097/01.meg.0000059107.41030.c7 12702914

